# Effect of itaconic acid production on *Neurospora crassa* in consolidated bioprocessing of cellulose

**DOI:** 10.1186/s12934-023-02034-0

**Published:** 2023-02-11

**Authors:** Jiajia Zhao, Caihong Ma, Yaojie Mei, Jingjing Han, Chen Zhao

**Affiliations:** 1 College of Life Sciences, Northwest A&F University, 22 Xinong Road, Yangling, 712100 Shaanxi China; 2 Biomass Energy Center for Arid and Semi-arid Lands, Northwest A&F University, 22 Xinong Road, Yangling, 712100 Shaanxi China

**Keywords:** Consolidated bioprocessing, *Neurospora crassa*, Itaconic acid, Cellulase, Lignocellulose

## Abstract

A system for itaconic acid synthesis from cellulose by *Neurospora crassa* was established, resulting in the highest yield of itaconic acid was 354.08 + 35.99 mg/L. Meanwhile, cellulase activity increased significantly, without any strain modifications for improved cellulase production. Multi-omics analyses showed that itaconic acid synthesis reduced energy production, leading to decreases in trehalose, cell wall, fatty acids synthesis and downregulations in MAPK signaling pathway, cell cycle and meiosis. More importantly, the low-energy environment enhanced the energy-efficient cellobionic acid/gluconic acid pathway, and the cellulase composition also changed significantly, manifested as the up-regulation of LPMOs and the down-regulation of β-glucosidases. Enhancing LPMOs-cellobionic acid/gluconic acid system has the potential to reduce energy consumption of the consolidated bioprocessing. These findings offer an overview of resource allocations by *N. crassa* in response to itaconic acid synthesis and highlight a series of intriguing connections between itaconic acid synthesis and cellulase synthesis in consolidated bioprocessing.

**Supplementary Information:**

The online version contains supplementary material available at 10.1186/s12934-023-02034-0.

## Background

Itaconic acid has a wide range of applications in industries such as resins, fibers, detergents and bioactive components, and is elected as the twelve high value-added chemical monomers [1]. The current world market for itaconic acid is estimated at $216 million [2]. In industry, itaconic acid is mainly produced by *Aspergillus terreus* from carbohydrate substrates, but its production cost is still higher than those of petrochemical products. Using lignocellulose as the raw material to produce itaconic acid may be one of the ways to reduce the production cost. Lignocellulose has been considered as the potential alternative to fossil resources in the production of chemicals and fuels for a long time [3]. At present, the conversion of lignocellulose is expensive and inefficient, and the conventional bioprocess includes cellulase production, saccharification of polysaccharides (cellulose and hemicellulose), and fermentation of monosaccharides (hexose and pentose), respectively. In contrast, consolidated bioprocessing (CBP) is simpler because it is carried out in a single container, avoiding the need for cellulase production and enzymatic hydrolysis steps [4]. However, the CBP involves the production of cellulase and the synthesis of target metabolites, which is a very complex system. A better understanding of the relationship between the synthesis of cellulase and itaconic acid will help to improve this CBP.

*N. crassa* is a natural cellulose decomposing fungus, and it is predicted to contain twice as many cellulases in its genome as *Trichoderma reesei* [5, 6]. It grows fast and is able to use a variety of carbon sources. Most importantly, it enables rapid use of glucose and xylose which are important monosaccharides in lignocellulose [7]. In addition, *N. crassa* has established mature research and operation methods in genetics, biochemistry, molecular biology and systems biology [8, 9], and the safety of its academic and commercial applications has also been proven [10]. Hence, *N. crassa* is a good organism for studying CBP. Wild-type *N. crassa* does not secret itaconic acid, however, the secretion of itaconic acid can be realized by expressing *A. terreus cis*-aconitic acid decarboxylase (cad1). In *A. terreus*, itaconic acid was synthetized by cad1 using *cis*-aconitic acid as precursor [11]. *Cis*-aconitic acid in the fungal mitochondrial TCA cycle is transported to the cytoplasm by mitochondrial transporter mttA, where *cis*-aconitic acid is decarboxylated to produce itaconic acid [12]. The transporter mttA plays an important role in efficient biosynthesis of itaconic acid [13].

In this study, *A. terreus* cad1 and mttA were used to construct the synthesis pathway of itaconic acid in *N. crassa* firstly. At the same time, we found that during the synthesis of itaconic acid, the recombinant *N. crassa*'s ability to secrete cellulase also increased. Then, how itaconic acid production could influence the carbon metabolic pathway and how it could regulate the gene expression levels of cellulase were investigated by metabolomics and transcriptomics analyses. We found that some key pathways and cellulase components affected by itaconic acid synthesis, and elucidated the relationship between itaconic acid synthesis and cellulase synthesis. Our findings will contribute to a deeper understanding of the fundamental mechanism of CBP.

## Results

### Construction of the *N. crassa* recombinants to produce itaconic acid

**Fig. 1:**
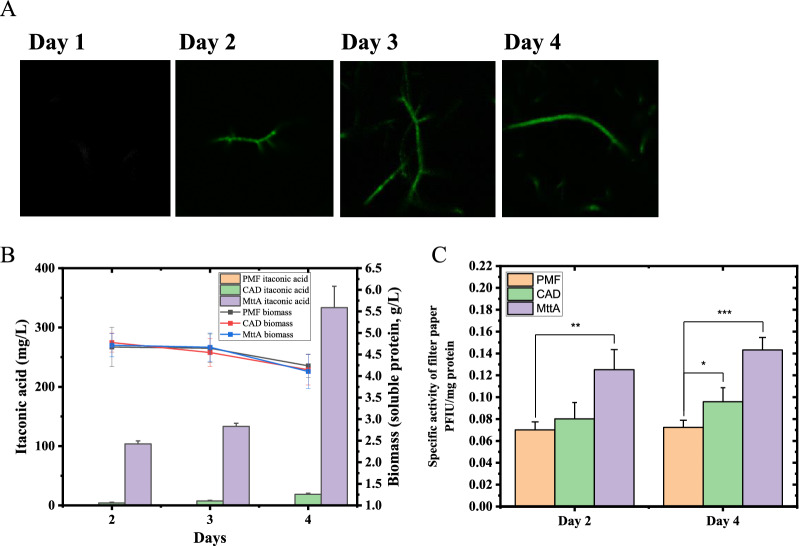
**A** Green fluorescence images of the mttA::GFP fusion expressed strain on Day 1, Day 2, Day 3 and Day 4. **B** Itaconic acid and biomass productions of the strain PMF, CAD and MttA on different culture phases with Avicel as the substrate. **C** Specific activities of filter paper (SAFP) of the strain PMF, CAD and MttA on Day 2 and Day 4, respectively. *T*-tests were conducted to evaluate statistical significance at* p* < 0.05(*), *p* < 0.01(**) and *p* < 0.001(***)

In order to construct the itaconic acid synthesis pathway in *N. crassa*, cad1 expressing vector pMF-272-cad1, and cad1 as well as mttA co-expressing vector pMF-272-cad1-mttA were constructed according to the steps described in the Materials and methods (Additional file 1: Fig. S1). As shown in Additional file 1: Fig. S2A, the amplification of the two vectors using cad1 primers resulted in bands of ~ 1500 bp, and the amplification of pMF-272-cad1-mttA using mttA and *Pcbh-1* primers resulted in bands of ~ 1000 bp (Additional file 1: Table S1), suggesting that these two vectors were successfully constructed, and their correctness were further verified by gene sequencing. Then, the two vectors and the control pMF-272 were transformed into *N. crassa* 9720, respectively, resulting in recombinant strains *N. crassa* 9720-pMF272-cad1 (CAD), 9720-pMF272-cad1-mttA (MttA) and 9720-pMF272 (PMF), and they were verified by PCR using the cad1 primers, mttA primers and pmf primers, respectively (Additional file 1: Table S1). The results are shown in Additional file 1: Fig. S2B. In order to test the expression of mttA in *N. crassa*, the strain that linked green fluorescent protein (GFP) to mttA was constructed (Additional file 1: Fig. S2B), and was imaged by a laser confocal microscopy. The images on different fermentation times are presented in Fig. 1A, which determined that mttA from *A. terraeus* was expressed in *N. crassa* on Day 2, and kept being synthesized till the end of fermentation on Day 4. The itaconic acid production by the strains CAD and MttA are shown in Fig. 1B. The itaconic acid yield of the strain CAD with the heterologous cad1 was 18.68 + 2.01 mg/L on Day 4 by shaking flask fermentation with Avicel as substrate, and the yield was improved by co-expression cad1 and mttA, which resulted in 354.08 ± 35.99 mg/L itaconic acid on Day 4 that was increased by 18 folds. There was no significant difference in the growth of the three strains, but their biomass decreased slightly on Day 4 (Fig. 1B). It's noteworthy that the specific activities of filter paper (SAFP, total cellulase activity per mg extracellular protein) were increased significantly in CAD on Day 4 and in MttA on both Day 2 and Day 4 (Fig. 1C). In particular, the SAFP of the strain MttA increased by 1.98 times compared with the control PMF on Day 4. However, nothing artificial optimization has be done for the synthesis of extracellular cellulase in this study. Therefore, we speculated that the synthesis of itaconic acid and cellulase may be closely regulated, and the strain reshaped its own regulatory mechanism under the pressure of itaconic acid synthesis. Then, the mechanism was further explored in the following multi-omics analyses.

### Effect of itaconic acid production on metabolome changes

The metabolomics analysis was carried out in order to analyze the effect of itaconic acid synthesis on *N. crassa* metabolism. The samples of CAD, MttA and the control PMF were analyzed by GC-TOF-MS. A total of 602 peaks were detected, of which 204 metabolites were analyzed after removing unknown peaks and repeated peaks. Principal Component Analysis (PCA) examined the overall distribution among CAD, MttA and PMF in Day 2 and Day 4, respectively (Additional file 1: Fig. S3A). It is shown that all the MttA groups and the CAD groups were clearly separated from the control groups except for the CAD groups on Day 2, which had a lower yield of itaconic acid. Comparative metabolome analysis showed that there were 14 (CAD vs PMF) and 22 (MttA vs PMF) metabolites improved by itaconic acid production on Day 2, while 11 (CAD vs PMF) and 20 (MttA vs PMF) metabolites were inhibited. However, levels of 60 (CAD vs PMF) and 85 (MttA vs PMF) metabolites increased on Day 4, while 3 (CAD vs PMF) and 13 (MttA vs PMF) metabolites decreased (Additional file 1: Fig. S3B). There were 11 common metabolites in the CAD vs PMF and MttA vs PMF on Day 2, and the unique metabolites increased to 47 on Day 4. Moreover, levels of aconitic acid, itaconic acid and 3,6-anhydro-D-galactose were significantly changed in all comparisons (Additional file 1: Table S2).

**Fig. 2:**
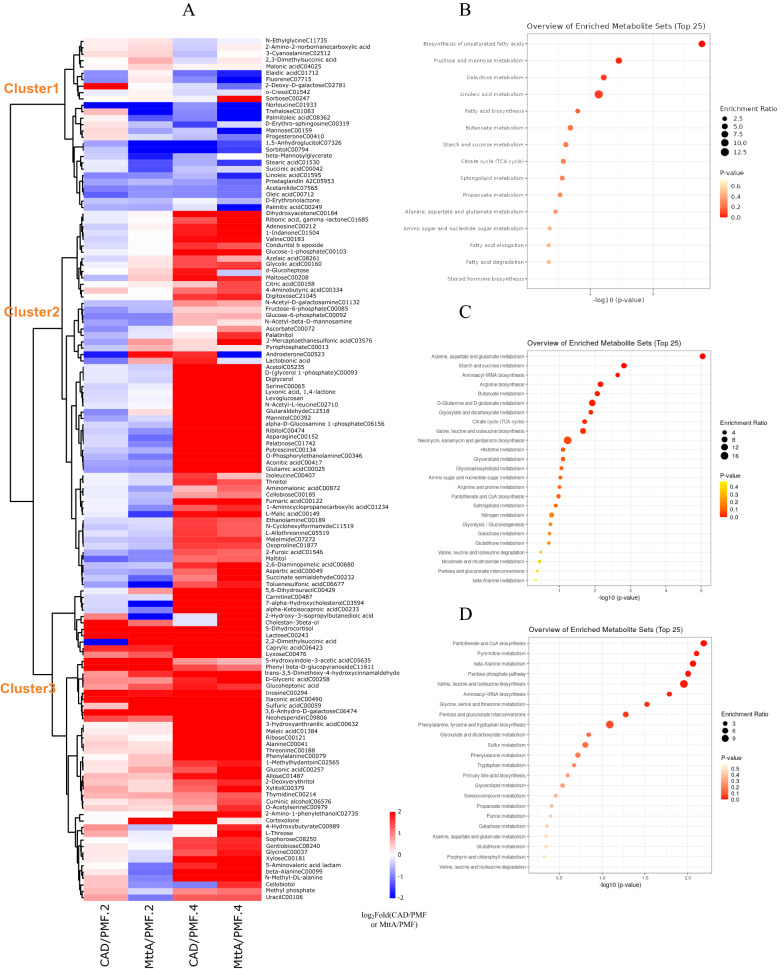

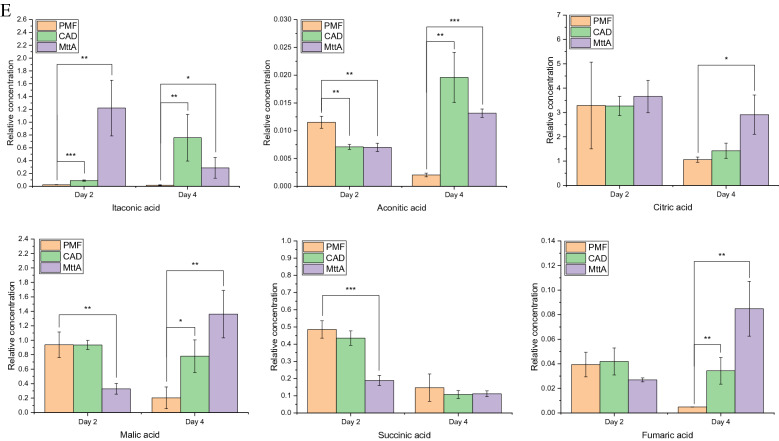
**A** Clustered heatmap profile of differential metabolites in CAD vs PMF and MttA vs PMF on Day 2 and Day 4. Similarity assessment for clustering was based on the canberra distance and word method. KEGG enrichment analysis for cluster 1 **B**, cluster 2 **C** and cluster 3 **D** in the clustered heatmap. **E** Intracellular concentrations of itaconic acid and other metabolites in the TCA cycle. *T*-tests were conducted to evaluate statistical significance at *p* < 0.05(*), *p* < 0.01(**) and* p* < 0.001(***)

According to the change of metabolite ratios, the metabolites with significant changes were divided into three groups for analysis (Fig. 2A). The metabolites in group 1 were mostly downregulated, and the main ways in which these metabolites involved were biosynthesis of unsaturated fatty acids, fructose and mannose metabolism, galactose metabolism and linoleic acid metabolism (Fig. 2B). Metabolites like trehalose, palmitoleic acid, mannose, stearic acid, linoleic acid, palmitic acid etc. were down-regulated more obviously in MttA vs PMF than in CAD vs PMF on Day 4. Different from the group 1, the metabolites on Day 4 in group 2 were mostly upregulated. The significantly changed metabolites belong to alanine, aspartate and glutamate metabolism, starch and sucrose metabolism, aminoacyl-rRNA biosynthesis, arginine biosynthesis as well as butanoate metabolism (Fig. 2C). In addition, metabolites related to itaconic acid precursors such as aconitic acid, malic acid, fumaric acid and so on were also in this group. Most metabolites of the group 3 were upregulated, and they were mainly involved in pantothenate and CoA biosynthesis, pyrimidine metabolism, β-alanine metabolism, pentose phosphate pathway as well as valine, leucine and isoleucine biosynthesis (Fig. 2D). Metabolites such as itaconic acid, 3,6-anhydro-D-galactose, lactose, etc. were most obviously up-regulated. It can be seen that the co-expression of cad1 and mttA led to a significant accumulation of itaconic acid in the cells, while the concentration of other TCA cycle intermediate metabolites, except citric acid, tended to decrease on Day 2 (Fig. 2E). However, the concentrations of aconitic acid, citric acid, malic acid and fumaric acid increased with itaconic acid synthesis after a period of cell adaptation on Day 4. This may be because the cellulase activity of MttA remained higher than that of the control group from Day 2 to Day 4, so it obtained more fermentable sugars during this period, making it overall metabolism more active. The vigorous metabolisms of CAD and MttA on Day 4 were also reflected in Fig. 2A. The above analysis indicated that the synthesis of itaconic acid had an effect on the metabolite levels of *N. crassa* on the second day of the fermentation, and the effect were further increased on the fourth day of fermentation.

### Effect of itaconic acid production on transcriptome changes

To further analyze the effect of itaconic acid production on cellulase, a transcriptional analysis of the high-yielding itaconic acid strain-MttA was performed on the second day of fermentation. The transcriptome profiles of MttA were compared with the control PMF. It was found 701 DEGs (differently expressed genes) in total, including 155 upregulated DEGs and 546 downregulated DEGs (Additional file 1: Fig. S4A). They were subjected to KOG (euKaryotic Ortholog Groups) and KEGG (Kyoto Encyclopedia of Genes and Genomes) enrichment analysis. According to the KOG enrichment analyses, the largest percentage of upregulated genes was related to amino acid transport and metabolism, and the downregulated genes was related to signal transduction mechanisms (Additional file 1: Fig. S4B). The results of the KEGG enrichment analysis showed that the most significantly upregulated DEGs were related to amino acids biosynthesis and metabolism, sulfur metabolism, pentose and glucuronate interconversions and 2-oxocarboxylic acid metabolism, and the downregulated DEGs were associated with oxidative phosphorylation and MAPK signaling pathway (Additional file 1: Fig. S4C).

**Fig. 3:**
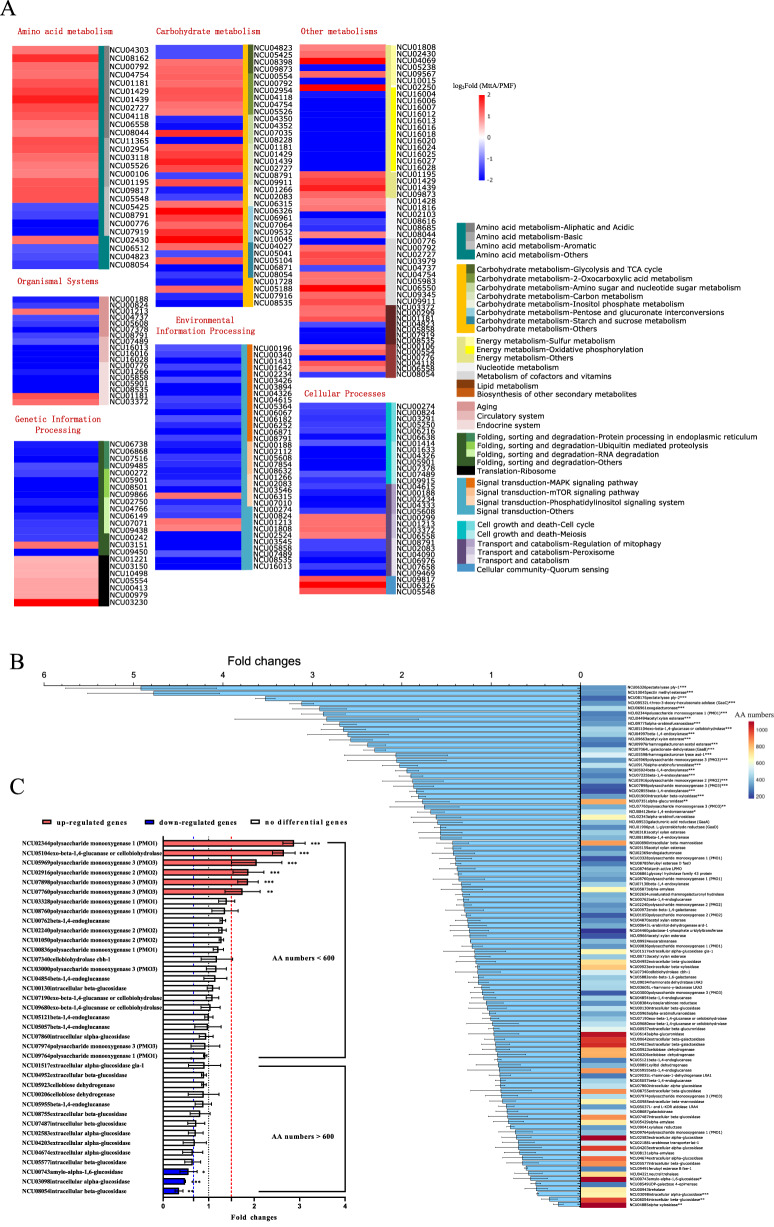
**A** Heatmap profile of DEGs from most annotated KEGG pathways in MttA vs PMF. **B** Fold changes of genes involved in plant biomass degradation. The heatmap reflected the number of amino acids in the enzyme encoded by the corresponding gene. **C** Fold changes in cellulase related gene expression. Two-way ANOVA by GraphPad Prism 9 was considered statistically significant at *p* < 0.05(*),* p* < 0.01(**) and *p* < 0.001(***)

All DEGs were further annotated with KEGG pathways (Fig. 3A). Most DEGs related to amino acid biosynthesis and metabolism were upregulated, mainly aliphatic, acidic and basic amino acid, such as NCU01429 and NCU01439 in glycine, serine, and threonine metabolism; NCU02727 and NCU06558 in glycine degradation; NCU04118 and NCU08162 in aspartic acid metabolism; NCU04754 in valine metabolism; NCU02954 and NCU03118 in lysine synthesis; NCU01195 in glutamate metabolism (See Fig. 5 for details). These amino acids have lower energetic costs than methionine, tyrosine and tryptophan [14, 15]. However, methionine synthase (NCU06512) related to methionine synthesis; tyrosinase (NCU00776) and catechol O-methyltransferase (NCU07919) related to tyrosine metabolism as well as catalase-1 (NCU08791) related to tryptophan metabolism were down-regulated, suggesting that the metabolism of tyrosine and tryptophan were inhibited.

For carbohydrate metabolism, the synthesis of itaconic acid negatively affected the expression of 2-oxoglutarate dehydrogenase (NCU05425) in TCA cycle. The DEGs in 2-oxocarboxylic acid metabolism as well as pentose and glucuronate interconversions were all upregulated. In amino sugar and nucleotide sugar metabolism, chitin synthase 5 and 6 (NCU04352, NCU04350) were downregulated, while glycosylhydrolase family 18–9 (NCU07035) associated with chitin degradation were upregulated.

For energy metabolism, most of DEGs were found to be associated with oxidative phosphorylation and sulfur metabolism. It clearly shows that almost all DEGs associated with oxidative phosphorylation metabolism were downregulated, such as NADH dehydrogenase subunit 1–6 (NCU16018, NCU16006, NCU16007, NCU16020, NCU16012 and NCU16004), cytochrome c oxidase subunit 1–2 (NCU16016, NCU16028), cytochrome b (NCU16013), ATPase subunit 6, 8, 9 (NCU16025, NCU16024, NCU16027). The upregulated DEGs in sulfur metabolism were linked to cytochrome c-1 (NCU01808), myo-inositol-1(or 4)-monophosphatase (NCU09567), 3′-phosphoadenosine 5′-phosphatase isoform B (NCU04069), while the downregulated DEGs were linked to methane sulfonate monooxygenase (NCU10015) and sulfite reductase beta subunit (NCU05238). Moreover, genes related to fatty acid synthesis were downregulated, such as NCU08535 and NCU05858, while those related to fatty acids degradation were upregulated, such as NCU00299 and NCU01181.

Unlike metabolism, most DEGs in organismal systems, environmental information processing, genetic information processing and cellular processes were downregulated. The DEGs observed in organismal systems mainly included aging, circulatory and endocrine system. The most significant pathway in signal transduction of the environmental information processing was MAPK signaling pathway with 15 DEGs downregulated (Additional file 1: Fig. S5). For genetic information processing, protein processing in endoplasmic reticulum, ubiquitin mediated proteolysis, RNA degradation and transport were found to be downregulated. For cellular processes, cell cycle, meiosis, regulation of mitophagy were downregulated, however, 4 DEGs related peroxisome were upregulated.

### Effect of itaconic acid production on cellulose degradation

The effect of itaconic acid on 101 genes involved in plant biomass degradation were analyzed (Fig. 3B) [16]. The upregulated genes mainly contained lytic polysaccharide monooxygenases (LPMOs), genes associated with galacturonide metabolism and xylan degradation. LPMOs are the fifth most abundant group of proteins in the cellulose secretome, next to endo-1,4-β-glucanase GH5-1, exo-1,4-β-glucanase CBH-1 and GH6-2, as well as β-glucosidase GH3-4 [17]. They are copper-dependent enzymes that cleave cellulose via oxidation. Three oxidation sites have been reported, including C1, C4 and C6 sites [18]. However, only genes related to oxidation at C1 and C4 sites were found in *N. crassa*. The upregulated LPMOs genes in the current study included *gh61-2* (NCU07760), *gh61-3* (NCU02916), *gh61-9* (NCU05969), *gh61-12* (NCU02344) and *gh61-13* (NCU07898). Among them, *gh61-12* is associated with oxidation at C1 site, *gh61-3* is associated with oxidation at C4 site, and *gh61-2* as well as *gh61-13* associated with oxidation at both C1 and C4 sites [19]. The galacturonide metabolism related genes involved NCU06326, NCU10045, NCU08176, NCU09532, NCU06961 and NCU07064, which are related to multistep reactions in pentose and glucuronate interconversions pathway (Fig. 3A). This pathway was also upregulated in the KEGG enrichment analysis (Additional file 1: Fig. S4C). The expressions of genes associated with xylan degradation were improved, such as NCU04997, NCU05924, NCU02855 and NCU04494. However, xylan was not added to the medium in this study. Znameroski et al. suggested that hemicellulases were partially regulated by cellulase [20]. Therefore, it may also be co-regulated with LPMOs in this study. It was worth noting that most of the enzymes encoded by these up-regulated genes had fewer amino acids, while most of the genes of enzymes containing more amino acids showed no change or downregulation (Fig. 3B). For example, in enzymes associated with cellulose degradation, the significant downregulated β-glucosidase (NCU08054) contains 980 amino acids, which is the highest number of amino acids among all seven β-glucosidases. The numbers of amino acids in these β-glucosidases are generally between 600 and 1000 (Fig. 3C). In contrast, the numbers of amino acids in upregulated LPMOs were only 238–369. This suggested that during the synthesis of itaconic acid, the strain preferred to synthesize enzymes related to biomass degradation with small molecular weights.

### Metabolic changes of LPMOs products

**Fig. 4:**
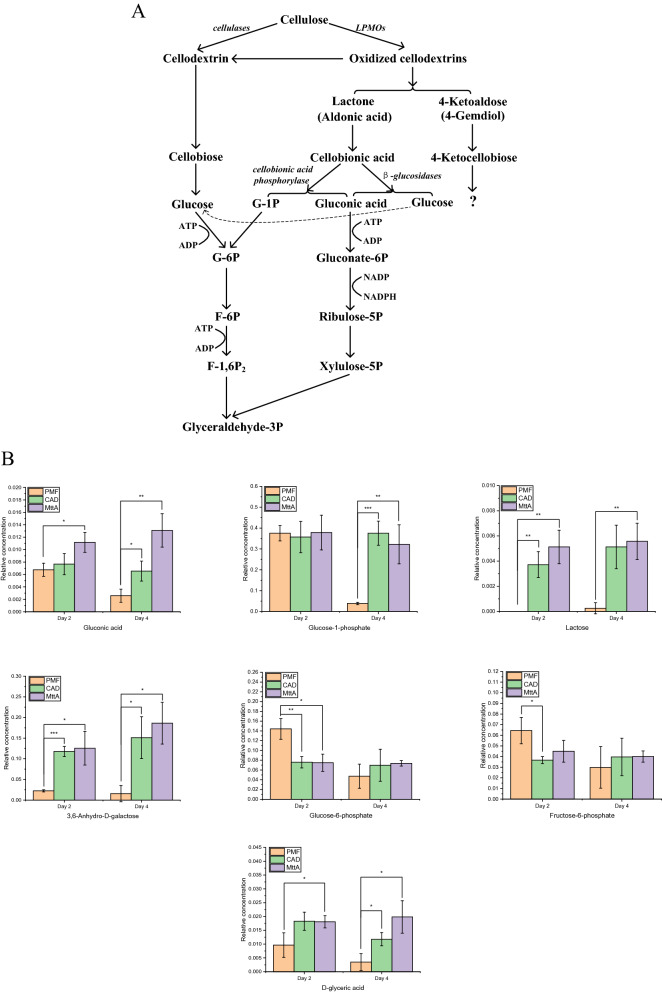
**A** Metabolic pathways of the cellulose degradants hydroxylated by LPMOs at C1 or C4 sites in *N. crassa.* **B** Intracellular concentrations of gluconic acid, glucose-1-phosphate, lactose, 3,6-anhydro-D-galactose, glucose-6-phosphate, fructose-6-phosphate and D-glyceric acid on Day 2 and Day 4 of PMF, CAD and MttA. *T*-tests were conducted to evaluate statistical significance at *p* < 0.05(*), *p* < 0.01(**) and *p* < 0.001(***)

Under the conditions of itaconic acid production, *N. crassa* tended to synthesize LPMOs which oxidized cellulose in C1 or C4 sites to generate oxidized cellodextrins. The product formed by the oxidation of C1 site is sugar lactone, which then further forms cellobionic acid and gluconic acid (Fig. 4A) [21, 22]. The cellobionic acid and gluconic acid enter the cytoplasm, and the former is catalyzed by cellobionic acid phosphorylase to produce gluconic acid and glucose-1-phosphate (G-1P) [22, 23]. Metabolite analysis showed that the relative concentration of gluconic acid increased gradually with itaconic acid production increasing (Fig. 4B). Generally, the gluconic acid is produced by intracellular glucose, however, the vital metabolites glucose-6-phosphate and fructose-6-phosphate in glycolysis pathway were downregulated on Day 2, and there was no significant increase on Day 4, indicating that the increase of gluconic acid was affected by LPMOs. Similarly. G-1P was significantly increased on Day 4, and the related lactose and galactose derivative 3,6-Anhydro-D-galactose were significantly upregulated. These results suggest that gluconic acid and G-1P were derived from other precursors besides glucose, such as sugar lactone formed from LPMOs oxidized at C1 site, and their contents were affected by the production of itaconic acid. Therefore, the promotion of LPMOs encouraged the metabolism of carbon sources through cellobionic acid and gluconic acid.

## Discussion

**Fig. 5:**
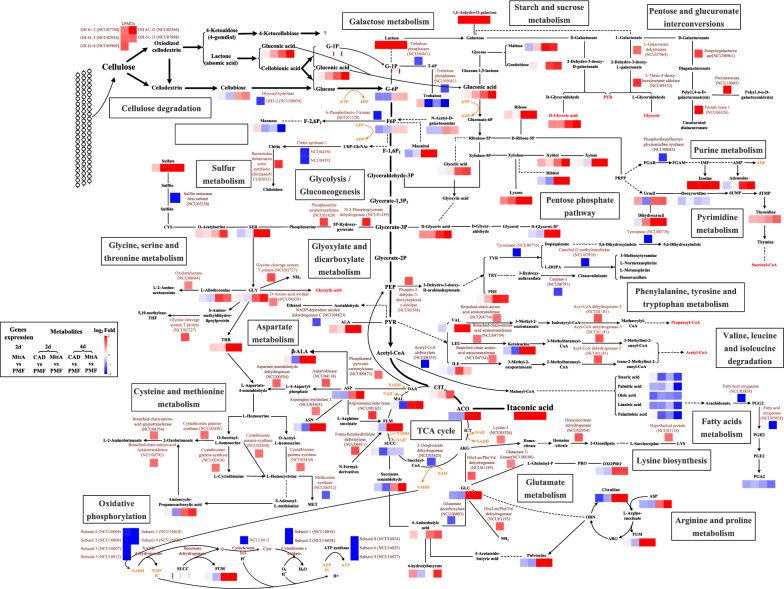
Network map of the itaconic acid synthesizing strain of *N. crassa* constructed based on comparative metabolome and transcriptome analysis data. The dotted line represents multi-step reactions, and the number of reactions is indicated on the dotted line. Metabolites are abbreviated as follows: G-1P, glucose-1-phosphate; G-6P, glucose-6-phosphate; F-6P, fructose-6-phosphate; F-1,6P_2_, fructose-1,6-bisphosphate, Gluconate-6P, gluconate-6-phosphate; Ribulose-5p, ribulose-5-phosphate; Xylulose-5P, xylulose-5-phosphate; L-Glutamyl-P, L-glutamyl 5-phosphate; Glyceraldehyde-3P, glyceraldehyde-3-phosphate; PEP, phosphoenol-pyruvate; PYR, pyruvic acid; T-6P, trehalose-6-phosphate; D-Glycerol-3P, D-glycerol-3-phosphate; CIT, citric acid; ACO, aconitic acid; ICT, isocitric acid; AKG, α-ketoglutaric acid; SUCC, succinic acid; FUM, fumaric acid; MAL, malic acid; OAA, oxaloacetic acid

In this study, the pathway to produce itaconic acid was established by the co-expression of *cad1* and *mttA* genes in *N. crassa.* Interestingly, the cellulase activity of the strain with higher yield of itaconic acid was also increased when cellulose was used as substrate. In order to investigate the effect of itaconic acid production on *N. crassa* metabolism and cellulase synthesis, the metabolomics and transcriptomics of itaconic acid-producing strains were analyzed, and the key information was summarized in a network map (Fig. 5).

On the second day of fermentation, itaconic acid was significantly accumulated in the strains of CAD and MttA. At this time, its precursor aconitic acid was downregulated. This reduced supply of precursors led to the reduction of other products in the TCA cycle such as succinic acid and malic acid in the strain MttA with higher-yield itaconic acid production. Its direct effect was the weakening of TCA cycle and oxidative phosphorylation process, manifested in the downregulation of 2-oxoglutarate dehydrogenase, NADH dehydrogenase, cytochrome C oxidase and ATP synthase. The strain adjusted itself under low energy conditions in many ways to keep the cellulase synthesis and itaconic acid synthesis ongoing.

### Increasing the expression of LPMOs while decreasing the expression of β-glucosidases

As mentioned before, cellulases with smaller molecular weights, such as LPMOs tend to be more highly expressed than larger ones, such as β-glucosidases (Fig. 3C). LPMOs act in synergy with cellulase and hemicellulose for hydrolyzing the lignocellulose more efficiently. β-glucosidase hydrolyzes cellooligosaccharides and cellobiose into glucose. The classical cellulase producing strain *T. reesei* accumulates cellobiose in hydrolysis process due to the lack of β-glucosidase components [24]. However, we did not find this phenomenon when cellulose was degraded by the recombinant *N. crassa* in this study. Compared with the control PMF, the β-glucosidase activity of MttA was significantly down-regulated on Day 4 (Additional file 1: Fig. S6), while the production of itaconic acid reached the maximum at this time, indicating that a moderate reduction of β-glucosidases expression level played a positive role in the production of itaconic acid in CBP.

### Metabolizing carbon sources through energy saving pathways

Compared to glucose, the metabolisms of cellobionic acid and gluconic acid are more energy efficient. Unlike the phosphorylation of glucose, the phosphorylation of the glucose residue of cellobionic acid does not require ATP [25]. One molecule of G-1P requires only one ATP to generate glycerate-3-phosphate through glycolysis. The gluconic acid is phosphorylated to glucose-6-phosphate, which is then metabolized to glyceraldehyde-3-phosphate through the pentose phosphate pathway also requiring one ATP per molecule. However, it takes 2 ATPs for each molecule of glucose metabolized to glyceraldehyde-3-phosphate (Fig. 5). This revealed that metabolizing cellulose in the form of cellobionic acid was favorable for cells to rapidly supplement carbon sources when energy supply for synthesis of itaconic acid was insufficient.

Oxidation at the C4 site yields 4-ketoaldose, followed by 4-ketocellobiose, but further metabolism is unknown [18]. The study by Beeson et al. showed that 4-ketoaldose can be converted to galactose in vitro which is a C4 epimer of glucose [26]. In this study, lactose and 3,6-Anhydro-d-galactose increased significantly on Day 2 and Day 4, suggesting that the conversion of 4-ketoaldose to galactose may also occur in *N. crassa*. The lactose and 3,6-Anhydro-d-galactose accumulated in MtttA can be transformed into d- and L-galactonate for metabolism to generate pyruvic acid, d-glyceric acid and glycerol for carbon source utilization (Fig. 5), of which the contents of d-glyceric acid were improved significantly (Fig. 4B). This pathway avoids the consumption of ATP for fructose-1,6-bisphosphate metabolism in glycolysis, and is therefore a potential energy saving pathway.

Five genes such as L-galactonate dehydratase and L-threo-3-deoxy-hexulosonate aldolase in pentose and glucuronate interconversions were significantly upregulated. The research by Wu et al. [16] showed that these genes were also upregulated in *N. crassa* when using glucuronic acid, galacturonic acid or pectin as substrates compared to cellulose, however, these substrates were not added to the medium. One possible reason is that the increased intracellular glucuronic acid or galacturonic acid content caused the corresponding expression of these genes. Interestingly, Chen et al. found LPMOs can oxidize cellulose at C6 site to produce C6-hexodialdoses, and its further hydrolysis produces glucuronic acid and glucose [27]. Therefore, it is possible that the upregulated LPMOs in this study may also have the capacity of oxidative cellulase at C6 site, thereby increasing the concentration of glucuronic acid to influence the pentose and glucuronate interconversions. This further proved the possibility that cellobionic acid could be metabolized by G-1P, lactose and galactose and so on to produce pyruvic acid.

### Reducing synthesis of cell wall components and fatty acids

During the synthesis of itaconic acid, the synthesis of mannose reduced. In *N. crassa*, mannose is involved in the synthesis of cell wall in the form of mannopyranose, and its total content accounts for 11.5% of the total carbohydrate in the cell wall [28]. The other main component of cell wall is chitin, and genes for its synthesis were negatively affected by the synthesis of itaconic acid. Acetyl-CoA carboxylase, the key enzyme to synthesizing fatty acids, was down-regulated, which mainly affected the syntheses of long-chain saturated fatty acids and unsaturated fatty acids, as well as the metabolites associated with even longer carbon chain, such as arachidonate-based metabolites. Fatty acids are building blocks of fuel sources, membranes and signaling molecules [29]. The downregulation of cell wall and fatty acids synthesis during itaconic acid synthesis can be regarded as a strategy to save energy and carbon source in energy deficiency.

### Attenuating cellular processes and signal transduction

During itaconic acid synthesis, cells growth and death were slowed. Protein kinases play crucial roles in the regulation of cellular processes. Transcriptome analysis revealed that many protein kinases were downregulated. For example, the MAPK signaling pathway which contains a group of serine-threonine protein kinases was inhibited (Additional file 1: Fig. S5). MAPK signaling pathway reflects adaptive responses to extracellular and intracellular conditions by regulating basic cell functions [30]. In this study, the extracellular conditions of MttA did not change, but the changes were from intracellular regulation. In addition, two serine-threonine protein kinases encoded by NCU07378 and NCU06638 were inhibited (Fig. 3A). The protein kinase encoded by NCU07378 acts as a transcriptional brake to control the expression of cellulase-encoding genes in *N. crassa*, and the cellulase activity can be increased when NCU07378 is knocked out. The downregulation of these two genes is beneficial to the synthesis of cellulase [31]. Therefore, cells were likely to conserve energy by reducing the expression and synthesis of protein kinases associated with cellular processes and signal transduction on the second day of itaconic acid synthesis by MttA.

### Reducing some energy-consuming pathways and metabolites

Sulfite reductase was downregulated and sulfate content in cells increased. Sulfate is usually activated by coupling it with ATP to generate APS or PAPS to increase the reduction potential [32]. Under the condition of energy deficiency, the utilization of sulfur was affected and the synthesis of sulfur-related amino acid methionine was decreased by down-regulating methionine synthase. In addition to methionine, other high energy consuming amino acids were also affected by itaconic acid synthesis, such as the downregulation of tyrosine and tryptophan metabolism. However, the synthesis and metabolism of the low energy consuming amino acids like serine, glycine, aspartic acid, valine etc. were active (Fig. 5), and this was consistent with the active protein synthesis confirmed by the upregulation of ribosomal proteins (Fig. 3A). Therefore, only downregulation of these high energy consuming amino acids was more likely related to the condition of energy deficiency caused by itaconic acid synthesis. Amino acids, as components of proteins, are also important precursors for cellulase synthesis. In this study, cellulase synthesis remained increased, but due to the low energy environment, the adjustment of cellulase components may also affect the synthesis and metabolism of its component amino acids. During the synthesis of itaconic acid, the synthesis of trehalose was reduced. Trehalose is a storage carbohydrate in response to changes in environmental conditions such as pH, calcium and heat stresses [33]. In this study, the pH, ion concentration and temperature of the medium did not change significantly, and therefore the reduction of trehalose was intended to make more carbon sources available for itaconic acid synthesis.

### Possible process of cellular adaptation to itaconic acid synthesis using cellulose as substrate according to the multi-omics analyses

**Fig. 6:**
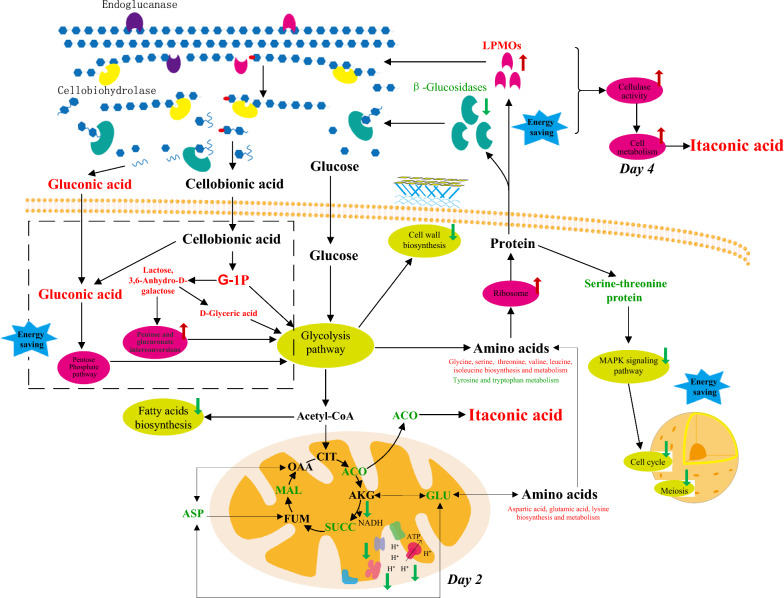
Schematic representation of the key regulation between itaconic acid and cellulase production by *N. crassa*. Red and green arrows or characters represent the upregulated and downregulated gene expression, metabolic process, enzyme activity or metabolite content, respectively. Metabolites are abbreviated as follows: G-1P, glucose-1-phosphate; CIT, citric acid; ACO, aconitic acid; ICT, isocitric acid; AKG, α-ketoglutaric acid; SUCC, succinic acid; FUM, fumaric acid; MAL, malic acid; OAA, oxaloacetic acid

Through the above discussion, the possible adaptation process of cells in synthetic itaconic acid is shown in Fig. 6**.** At the beginning of itaconic acid accumulation stage (Day 2), the TCA cycle and oxidative phosphate pathway were weakened. In response to cellulase synthesis and itaconic acid production, cells utilized more cellobiomic acid and gluconic acid for metabolism from the perspective of energy saving. Because these two metabolites are enzymatic products of LPMOs, their rapid depletion also promoted LPMOs synthesis, manifested by increased LPMOs expression in the cellulases. On the other hand, in a low-energy environment, the cells reduced the fatty acids, cell wall synthesis, and attenuated the MAPK signaling pathways, the cell cycle as well as the meiosis. After cell and metabolic regulations on Day 2, CAD and MttA cellulase activities remained high on Day 4 and the itaconic acid accumulation was also reached the highest level. Because the cellulase activity of itaconic acid-producing strains was higher than that of the control for a long time, their internal metabolisms were more active, manifested by the slightly increased cellobiose and glucose-6-phosphate, and the significantly increased intermediate metabolites in TCA cycle as well as other metabolic pathways shown in Fig. 5. Therefore, the simultaneous elevation of itaconic acid and cellulase activity benefited from overall cell regulation under low-energy conditions.

### Strategies for future construction of CBP strain

The conversion of cellulose to itaconic acid is divided into two processes which are actually complicated. For example, cellulose degradation involves multiple cellulase components and their synergistic effects. The synthesis of itaconic acid involves the complex regulation of central metabolic TCA cycle and the transport of mitochondria and cytoplasmic metabolites. In the process of analyzing the itaconic acid synthetic strain in this study, it was found that these two processes were co-regulated by the influence of energy. The overall synthesis efficiency of cellulase should be emphasized in the molecular optimization of cellulase system. Increasing the proportion of LPMOs in CBP system may helpful to improve the overall cellulase activity, and more importantly, LPMOs meets the energy saving requirements of CBP system due to its smaller molecular weight and lower metabolic energy consumption of enzymatic hydrolysis products. Carbon sources can be utilized by enhancing the cellobionic acid/gluconic acid pathway through metabolic engineering to promote the rapid utilization of the specific enzymatic hydrolysis products of LPMOs on cellulose. This enhanced LPMOs-cellobionic acid/gluconic acid system has lower energy consumption than the traditional cellulases-glucose metabolism system. On the other hand, reducing fatty acid synthesis, or attenuating cellular processes by regulated protein kinases such as NCU07378 and NCU06638, may also be effective ways to save energy.

## Conclusion

In this study, a synthetic pathway of itaconic acid was constructed in *N. crassa* by expressing cad1 and mttA, enabling it to produce itaconic acid from Avicel. It was found that the activity of cellulase improved with the increase of itaconic acid content, suggesting that metabolic changes affected the synthesis of cellulase. Multi-omics analyses revealed a global landscape of resource allocation by the genetically engineered *N. crassa* in response to energy deficiency caused by the itaconic acid synthesis, and the relationship between such allocation and cellulase synthesis. It was showed that the itaconic acid-producing strain increased the expression of LPMOs and reduced the expression of β-glucosidases in response to cellulose degradation in the case of energy reduction. Meantime, the related metabolism of gluconic acid and glucose-1 phosphate, the products of LPMOs, were also enhanced. The LPMOs-cellobionic acid/gluconic acid system has the potential to reduce energy consumption of CBP, providing reference for rational design of cellulase components and minimal system design for cellulose degradation to itaconic acid synthesis in the future.

## Materials and methods

### Strains, vector and strain construction

*N. crassa* strain FGSC 9720 (mus-52::bar, *his-3* mutant, mat A), wildtype FGSC 2489 and vector pMF-272 (*Pccg-1*, Ampicillin, *his*^+^) carrying green fluorescent protein were purchased from the Fungal Genetics Stock Center (FGSC). *Escherichia coli* strain DH5α was used as a host for plasmid proliferation. The *N. crassa* strain FGSC 9720 was used as a host for target gene expression. The vector pMF-272 (Additional file 1: Fig. S1A) was the fundamental framework of the construction of vectors. The gene encoding mitochondrial organic acid transporter (mttA, 906 bp, GenBank ID: HG423568.1) and *cis*-aconitic acid decarboxylase (cad1, 1473 bp, GenBank ID: AB326105.1) from *Aspergillus terreus* were codon optimized and synthesized by Sangon Biotech (Shanghai, China). The promoter *Pccg-1* was used to express cad1 to obtain vector pMF-272-cad1. The promoter *Pccg-1* and *Pcbh-1* were selected to express cad1 and mttA respectively to obtain vector pMF-272-cad1-mttA. The promoter *Pcbh-1* was cloned from 920 bp upstream of *N. crassa cbh1* gene. The method of seamless cloning (Ready-to-Use Seamless Cloning Kit by Sangon Biotech) was used to vector construction. The synthesized gene *cad1* was cloned into EcoR I + Xba I-digested vector pMF-272 to obtain vector pMF-272-cad1 (Additional file 1: Fig. S1B). Then, the synthesized gene *mttA* and *Pcbh-1* were cloned into Not I-digested vector pMF-272-cad1 to obtain vector pMF-272-cad1-mttA (Additional file 1: Fig. S1C). For verifying mttA expression, the synthesized gene *mttA* was cloned into Pac I + Xba I-digested vector pMF-272 to obtain vector pMF-272-mttA::GFP (Additional file 1: Fig. S1D). Primers used for vector construction are shown in Additional file 1: Table S3. All vectors were transformed into *N. crassa* FGSC 9720 by electrotransformation [34]. The pMF-272 with histidine expression cassette was also transformed into *N. crassa* 9720 to compensate for its histidine deficiency, and the obtained transformant was used as control strain, so as to avoid the slow growth caused by histidine deficiency that may interfere with subsequent omics analysis. After screening positive transformants, the homokaryons were obtained by the method described by Ebbole et al. [35]. For screening and imaging, the hyphae were transferred to fermentation medium (2% (w/v) Avicel and 1 × Vogel’ salts [36]) under dark condition for 48 h, of which small amount were placed onto a microscope slide. The hyphae were immediately imaged using a dualchannel photomultiplier tube confocal system (CLSD-2SS, Thorlabs) on a Leica DMi8 microscope. Samples were excited by a laser at 488 nm using a 1.4 NA × 100 oil immersion objective and fluorescence detected at 497-531 nm.

### Media and culture conditions

The media used for *N. crassa* growth and molecular manipulation were detailed in many studies [34, 37, 38]. For conidia harvest, *N. crassa* cells were inoculated in 250 ml shaker with1.5% agar, 2% (w/v) sucrose and 1 × Vogel’s salts [36] and grown under dark condition for 3 days at 30 °C followed by light condition for 7 days at 25 °C. When a large number of conidia were formed, they were collected by adding 25 ml distilled water to the shaker and filtering through eightfold cheesecloth. The conidia (1 × 10^6^/mL final concentration) were then inoculated into seed medium containing 2% (w/v) sucrose and 1 × Vogel’s salts and cultured at 28℃ under dark condition for 40 h. The hyphae were collected into fermentation medium containing 2% (w/v) Avicel and 1 × Vogel’s salts for 96 h at 28℃ under dark conditions and a shaking of 220 rpm.

### Metabolite extraction and derivatization

For the intracellular metabolite analysis, the hyphae were collected by rapid filtration and stored in liquid nitrogen. 25 ± 1 mg sample was transferred into a 2 mL EP tube, and 220 μL pure water were added. Then the sample with steel ball added was processed with 35 Hz grinding instrument for 4 min, and ultrasonic treatment with ice water bath for 5 min. The process was repeated for three times. Appropriate amount (about 1000 μL) of pre-cooled methanol:chloroform (3:1) solvent was added in the 20 μL of the above cell mixture according to the protein concentration of it. Five microliters adonitol were added as internal standard. The sample was vortexed for 3 min and centrifuged at 4 ℃ (RCF = 13800 (× g), R = 8.6 cm) after ultrasonic treatment in ice water bath for 10 min. The supernatant of 450 μL was moved into 1.5 mL EP tube and the sample extract was dried using a vacuum concentrator. For metabolite derivatization, 40 μL of methoxyamination hydrochloride (20 mg/mL in pyridine) was added to the dried extract and incubated at 80 ℃ for 30 min. Then 50 μL of BSTFA regent (1% TMCS, v/v) was added and incubated at 70 ℃ for 1.5 h.

### RNA isolation

Total RNA was extracted using the Total RNA Extractor (Trizol) kit (Sangon, China) according to the manufacturer’s protocol, and treated with RNase-free DNase I to remove genomic DNA contamination. RNA integrity was evaluated with a 1.0% agarose gel. The quality and quantity of RNA were assessed using a Qubit^®^ RNA Assay Kit in Qubit^®^2.0 Flurometer (Life Technologies, CA). The RNA samples with high quality were subsequently submitted to the Sangon Biotech (Shanghai, China) Co., Ltd. for library preparation and sequencing. A total amount of 2 μg RNA per sample was used as input material for the RNA sample preparations.

### Analysis methods

The derivatized metabolite samples were analyzed by an Agilent 7890 gas chromatograph coupled with a time-of-flight mass spectrometer. The system was equipped with a DB-5MS capillary column. Helium was used as the carrier gas. The front inlet purge flow was 3 mL/min, and the gas flow rate through the column was 1 mL/min. The inlet, interface, and ion source temperatures were 280, 280 and 250 °C, respectively. One microliter aliquot of sample was injected in splitless mode. The initial temperature was kept at 50 °C for 1 min, then raised to 310 °C at a rate of 10 °C/min, and held at this temperature for 8 min. The energy was − 70 eV in electron impact mode. The mass spectrometry data were acquired in full-scan mode with the m/z range of 50–500 at a rate of 12.5 spectra per second after a solvent delay of 6.2 min. Raw data analysis was finished with Chroma TOF (V 4.3x, LECO) software and LECO-Fiehn Rtx5 database was used for metabolite identification by matching the mass spectrum and retention index [39]. The metabolome experiment was performed in three parallels. In general, the metabolites with the threshold of *P* value < 0.05, and |log_2_FC|> 1 were considered as differential metabolites. Principal Component Analysis and other statistical analysis were conducted using the Metaboanalyst online service (http://www.metaboanalyst.ca/faces/home.xhtml). The metabolic pathway information was extracted from KEGG.

Sequencing libraries were generated using VAHTSTM mRNA-seq V2 Library Prep Kit for Illumina^®^ following manufacturer’s recommendations, and the library quality was assessed on the Agilent Bioanalyzer 2100 system. Paired-end sequencing of the library was performed on the HiSeq XTen sequencers (Illumina, San Diego, CA). FastQC (version 0.11.2) was used for evaluating the quality of sequenced data. Raw reads were filtered by Trimmomatic (version 0.36). Clean reads were mapped to the *N. crassa* OR74A genome by HISAT2 (version 2.0) with default parameters. Gene expression values of the transcripts were computed by StringTie (version 1.3.3b). TPM (Transcripts Per Million) is a commonly used method for estimating gene expression levels, considering the effect of sequencing depth, gene length and sample on reads count. Differentially expressed genes between two samples were selected by DESeq2 (version 1.12.4) with the threshold of |log_2_FC|> 1 and *P* value < 0.05. KOG and KEGG pathway analysis were performed to identify which differentially expressed genes were significantly enriched in KOG functions or metabolic pathways.

The detection methods of filter paper activity (FPA) and β-glucosidase were determined according to the methods established by Ghose [40]. Bradford method was used to determine extracellular soluble protein content and the biomass protein content.

Extracellular itaconic acid was quantified by a Shimadzu LC-20A HPLC system equipped with a Bio-Rad Aminex HPX-87H column. The column was operated at 55 ℃ temperature, 0.6 mL/min flowrate, and 0.005 M H_2_SO_4_ as mobile phase. One milliliter culture sample was centrifuged, and 10 μL supernatant was detected with UV-vis absorption at 215 nm.

## Supplementary Information

**Additional file 1: Figure S1.** Profiles of pMF272, pMF-272-cad1, pMF-272-cad1-mttA and pMF-272- mttA::GFP. **Table S1.** Primers for gene identification. **Figure S2.** (A) PCR verification of the vector construction. M: marker; 1: cad1 gene in pMF-272-cad1; 2: cad1 gene in pMF-272-cad1-mttA; 3: mttA gene in pMF-272-cad1-mttA; 4: Pcbh-1 in pMF-272-cad1-mttA. (B) PCR verification of recombinant strains N. crassa 9720-pMF272-cad1 (CAD), 9720-pMF272-cad1-mttA (MttA) and 9720-pMF272 (PMF). M: marker; 1-3: cad1 gene in the strain CADs; 4-6: mttA gene in the strain MttAs; 7: Pccg-1+gfp in the strain PMF (~1600 bp); mttA+gfp in the strains of fusion expression of mttA and GFP (1626 bp). **Figure S3.** (A) Score visualizing of the Principal Component Analysis (PCA). (B) Volcano plots of differential metabolites in CAD vs PMF and MttA vs PMF on Day 2 and Day 4. Red and blue dots respectively denote the upregulated and downregulated differential metabolites in the CAD and MttA groups, compared with those in the PMF group. **Table S2.** Differential metabolites and common differential metabolites in CAD vs PMF and MttA vs PMF on Day 2 and Day 4. **Figure S4.** (A) Volcano plots of differently expressed genes in MttA vs PMF. Red and green dots respectively denote the upregulated and downregulated differential metabolites (P-value<0.05 and |log2FC|>1) in the MttA groups compared with those in the PMF group. KOG (B), and KEGG (C) enrichment analysis of the differently expressed genes in MttA vs PMF. **Figure S5.** Changes in expression levels of MAPK signaling pathway related genes in MttA compared with PMF. **Figure S6.** Comparison of β-glucosidase activities between PMF and MttA strains. T-tests were conducted to evaluate statistical significance at p < 0.05(*). **Table S3.** Primers in vector construction.

## Data Availability

All data generated or analyzed during this study are included in this article and its Additional file 1.

## Abbreviations

CBP: Consolidated bioprocessing
LPMOs: Lytic polysaccharide monooxygenases
cad1: *cis*-Aconitic acid decarboxylase
mttA: Mitochondrial organic acid transporter
SAFP: Specific activity of filter paper
KEGG: Kyoto encyclopedia of genes and genomes
PCA: Principal component analysis
DEGs: Differently expressed genes
GO: Gene ontology
KOG: Eukaryotic ortholog groups
G-1P: Glucose-1-phosphate
FGSC: Fungal genetics stock center
FPA: Filter paper activity
HPLC: High-performance liquid chromatography
