# Supplementary material for: Effect of itaconic acid production on Neurospora crassa in consolidated bioprocessing of cellulose
Source: Microb Cell Fact. 2023 Feb 11;22:28. doi: 10.1186/s12934-023-02034-0 (PMC9922455; doi:10.1186/s12934-023-02034-0)
Supplement: Supplementary file 1 — Additional file 1: Figure S1. Profiles of pMF272, pMF-272-cad1, pMF-272-cad1-mttA and pMF-272- mttA::GFP. Table S1. Primers for gene identification. Figure S2. (A) PCR verification of the vector construction. M: marker; 1: cad1 gene in pMF-272-cad1; 2: cad1 gene in pMF-272-cad1-mttA; 3: mttA gene in pMF-272-cad1-mttA; 4: Pcbh-1 in pMF-272-cad1-mttA. (B) PCR verification of recombinant strains N. crassa 9720-pMF272-cad1 (CAD), 9720-pMF272-cad1-mttA (MttA) and 9720-pMF272 (PMF). M: marker; 1-3: cad1 gene in the strain CADs; 4-6: mttA gene in the strain MttAs; 7: Pccg-1+gfp in the strain PMF (~1600 bp); mttA+gfp in the strains of fusion expression of mttA and GFP (1626 bp). Figure S3. (A) Score visualizing of the Principal Component Analysis (PCA). (B) Volcano plots of differential metabolites in CAD vs PMF and MttA vs PMF on Day 2 and Day 4. Red and blue dots respectively denote the upregulated and downregulated differential metabolites in the CAD and MttA groups, compared with those in the PMF group. Table S2. Differential metabolites and common differential metabolites in CAD vs PMF and MttA vs PMF on Day 2 and Day 4. Figure S4. (A) Volcano plots of differently expressed genes in MttA vs PMF. Red and green dots respectively denote the upregulated and downregulated differential metabolites (P-value<0.05 and |log2FC|>1) in the MttA groups compared with those in the PMF group. KOG (B), and KEGG (C) enrichment analysis of the differently expressed genes in MttA vs PMF. Figure S5. Changes in expression levels of MAPK signaling pathway related genes in MttA compared with PMF. Figure S6. Comparison of β-glucosidase activities between PMF and MttA strains. T-tests were conducted to evaluate statistical significance at p < 0.05(*). Table S3. Primers in vector construction. [file 12934_2023_2034_MOESM1_ESM.docx]

**Supplementary material for Publication**

Effect of itaconic acid production on *Neurospora crassa* in consolidated bioprocessing of cellulose

Jiajia Zhao^a,b^, Caihong Ma^a,b^, Yaojie Mei^a,b^, Jingjing Han^a,b^, Chen Zhao^a,b*^

^a^ College of Life Sciences, Northwest A&F University, 22 Xinong Road, Yangling 712100, Shaanxi, China

^b^ Biomass Energy Center for Arid and Semi-arid Lands, Northwest A&F University, 22 Xinong Road, Yangling 712100, Shaanxi, China

*Corresponding Author

Address: College of Life Sciences, Northwest A&F University, 22 Xinong Road, Yangling 712100, Shaanxi, China.

E-mail: zhaochencn@hotmail.com (C. Zhao).


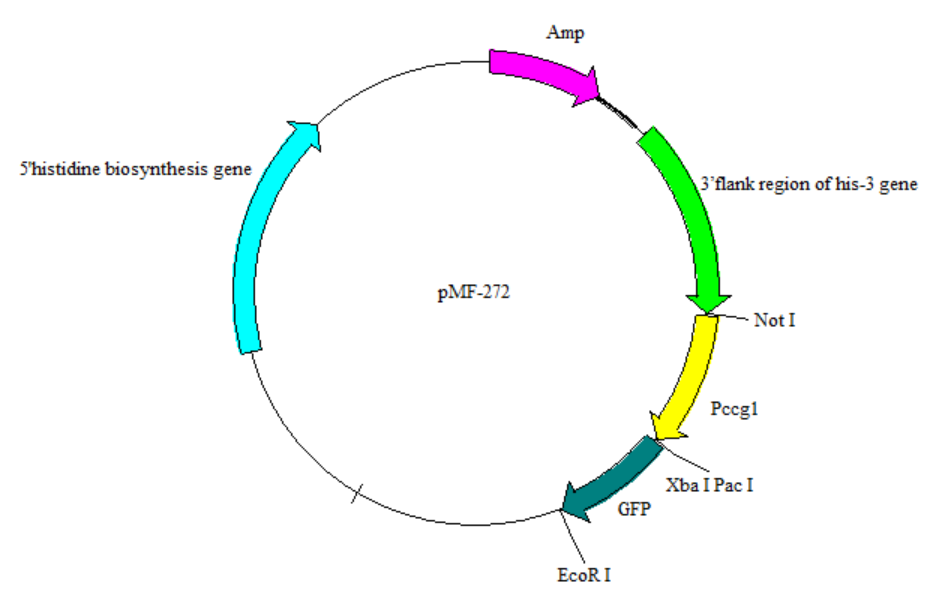

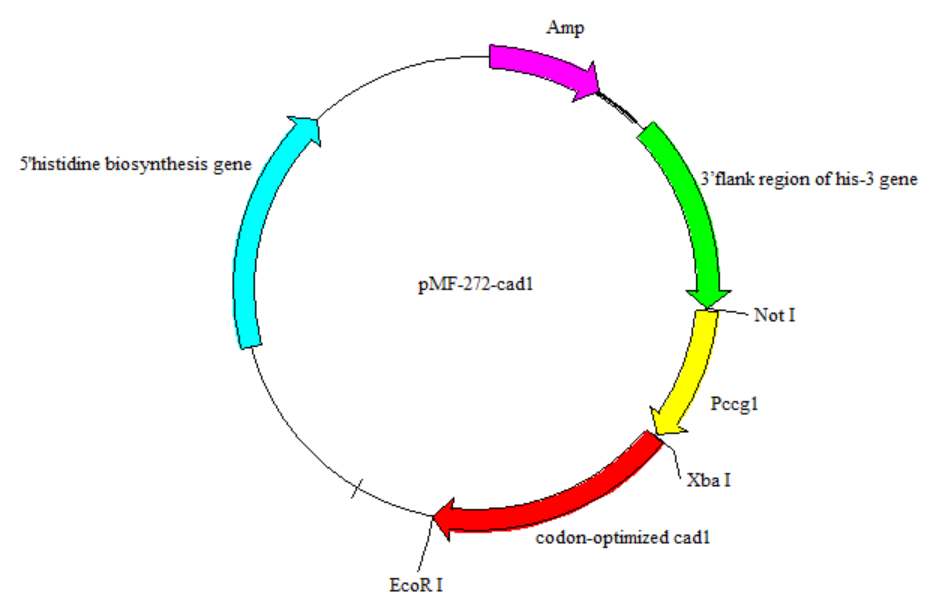

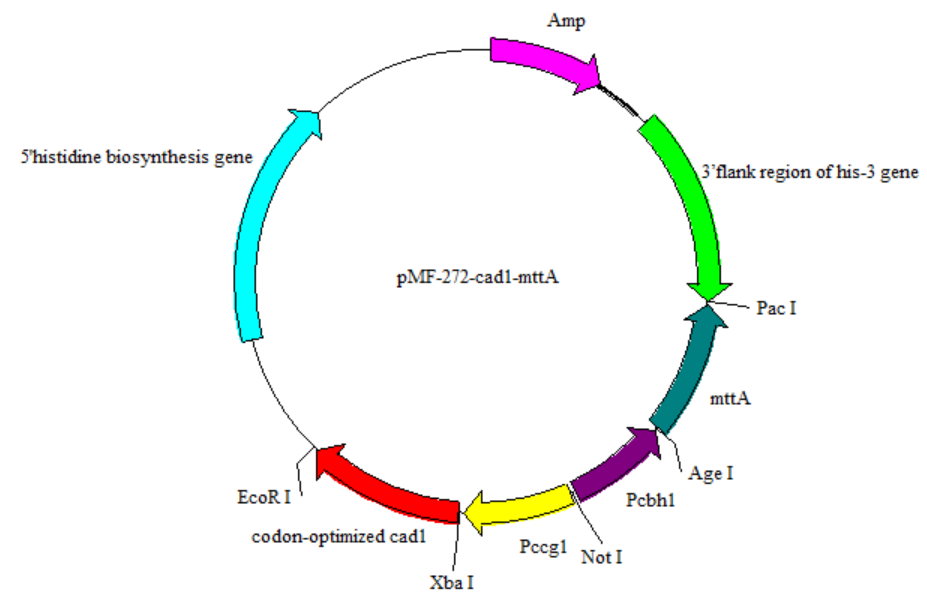

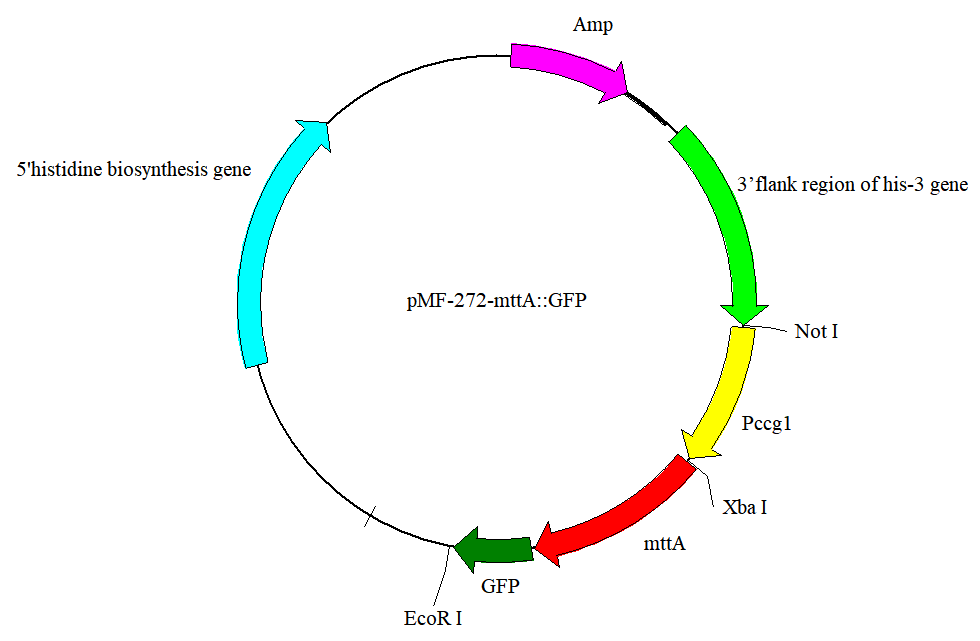


A

B

D

C

**Fig. S1.** Profiles of pMF272, pMF-272-cad1, pMF-272-cad1-mttA and pMF-272- mttA::GFP.

**Table S1.** Primers for gene identification.

| Name | Description |
| --- | --- |
| cad1-F | ATGACCAAGCAGTCCGCCGA |
| cad1-R | TCAAGAGCCCCCTCGTCTGA |
| Pcbh1-F | CTTGAAGCTGCCAACTCAACC |
| Pcbh1-R | GGTGAAGATGAGGCTGAACGG |
| mttA-F | ATGGATAGTAAGATCCAAAC |
| mttA-R | TCAGTTAGGCTGGGTGAGGA |
| pmf-F | TAGAAGGAGCAGTCCATCTG |
| pmf-R | TTACTTGTACAGCTCGTCCA |
| mttA::GFP-F | ATGGATAGTAAGATCCAAAC |
| mttA::GFP-R | TTACTTGTACAGCTCGTCCA |

A

B

**Fig. S2.** (A) PCR verification of the vector construction. M: marker; 1: *cad1* gene in pMF-272-cad1; 2: *cad1* gene in pMF-272-cad1-mttA; 3: *mttA* gene in pMF-272-cad1-mttA; 4: *Pcbh-1* in pMF-272-cad1-mttA. (B) PCR verification of recombinant strains *N. crassa* 9720-pMF272-cad1 (CAD), 9720-pMF272-cad1-mttA (MttA) and 9720-pMF272 (PMF). M: marker; 1-3: *cad1* gene in the strain CADs; 4-6: *mttA* gene in the strain MttAs; 7: *Pccg-1*+*gfp* in the strain PMF (~1600 bp); *mttA*+*gfp* in the strains of fusion expression of mttA and GFP (1626 bp).

A


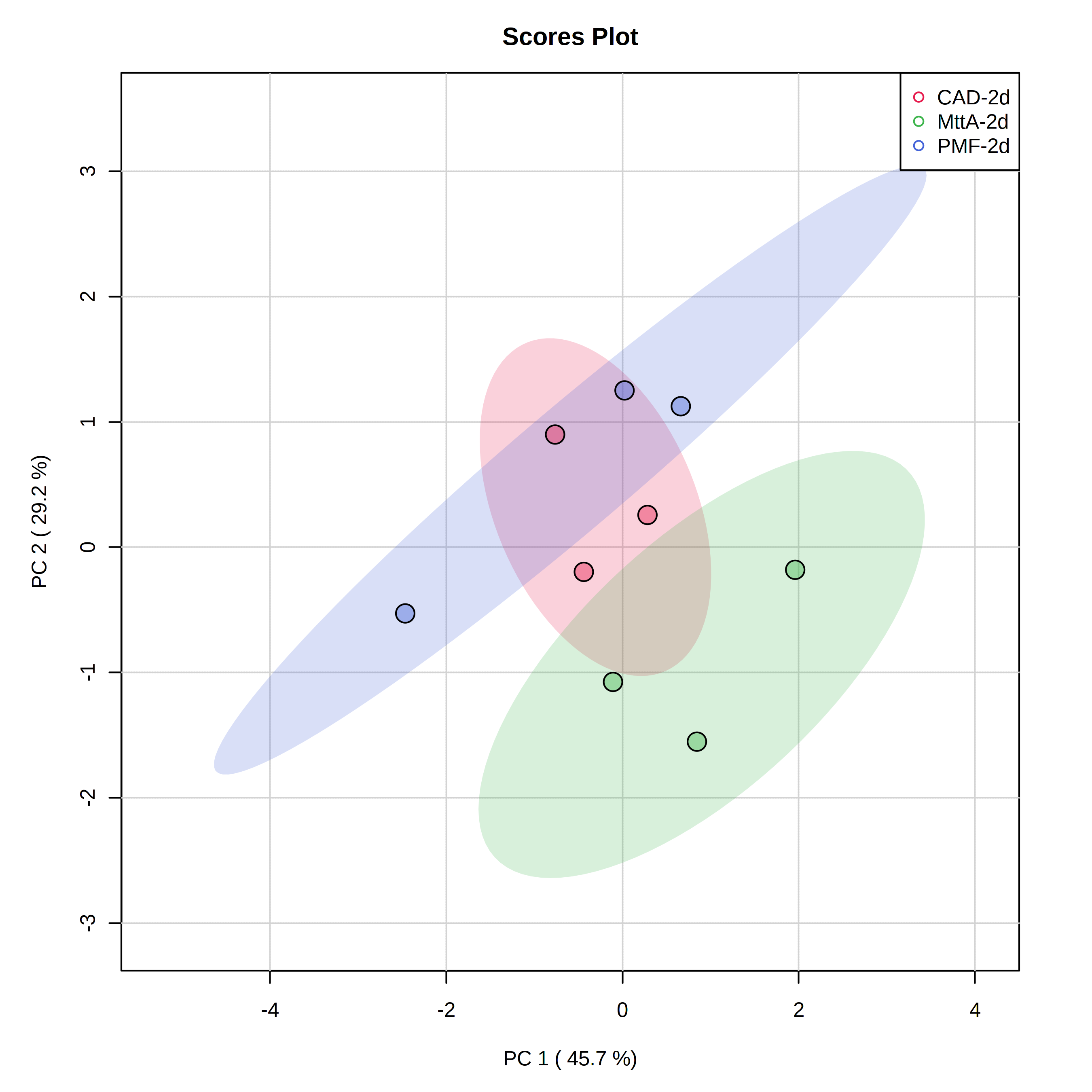

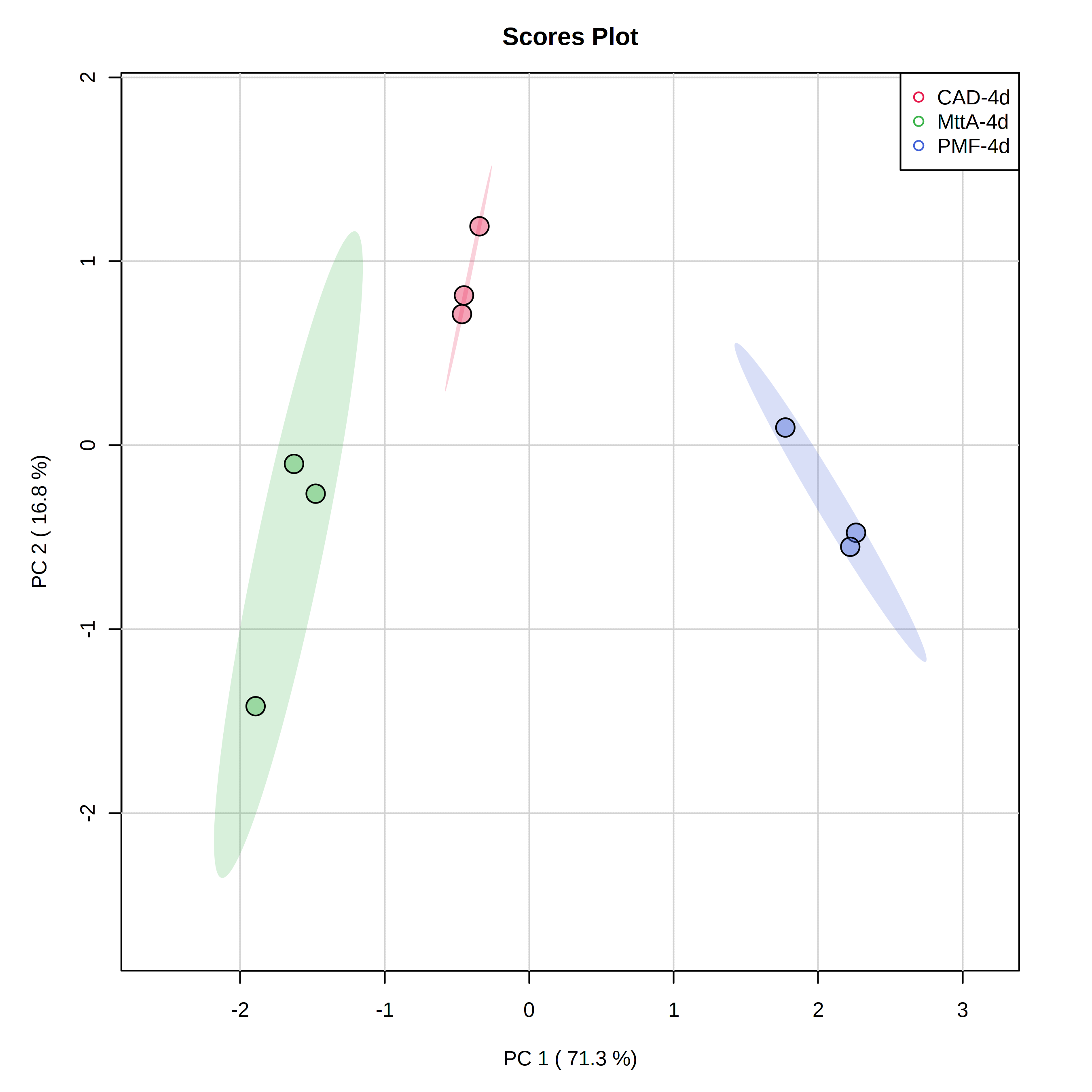


4d

2d

B


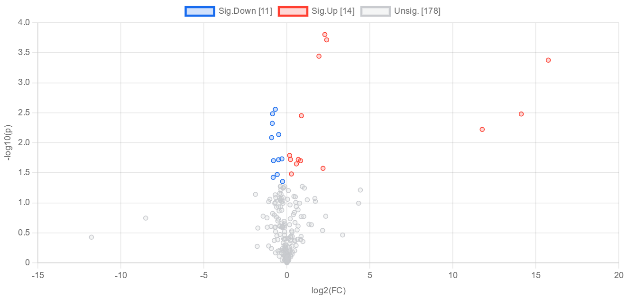

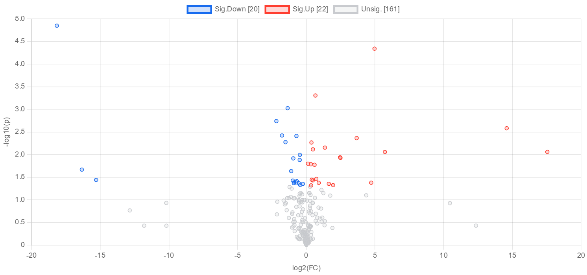


MttA VS PMF 2d

CAD VS PMF 2d


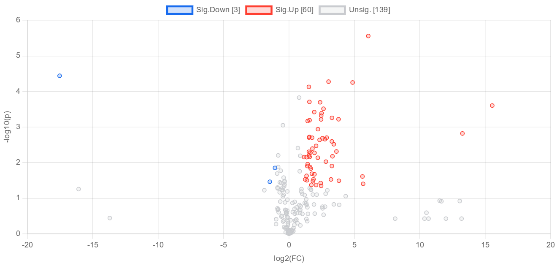

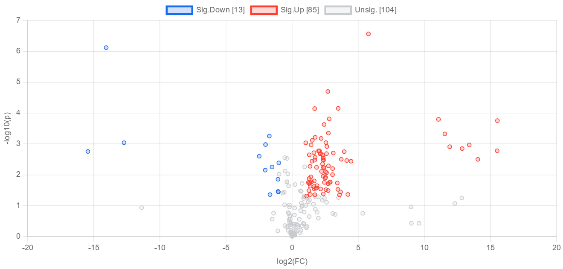


MttA VS PMF 4d

CAD VS PMF 4d

**Fig. S3.** (A) Score visualizing of the Principal Component Analysis (PCA). (B) Volcano plots of differential metabolites in CAD vs PMF and MttA vs PMF on Day 2 and Day 4. Red and blue dots respectively denote the upregulated and downregulated differential metabolites in the CAD and MttA groups, compared with those in the PMF group.

**Table S2.** Differential metabolites and common differential metabolites in CAD vs PMF and MttA vs PMF on Day 2 and Day 4.

| **CAD vs PMF, Day 2** | **MttA vs PMF, Day 2** |
| --- | --- |
| 2-Deoxy-D-galactose | 1,5-Anhydroglucitol |
| 3,6-Anhydro-D-galactose **#** | 2,2-Dimethylsuccinic acid |
| 3-Cyanoalanine # | 2,3-Dimethylsuccinic acid |
| 4-Hydroxybutyrate # | 2-Amino-2-norbornanecarboxylic acid |
| 5-Dihydrocortisol | 2-Furoic acid |
| Aconitic acid **#** | 3,6-Anhydro-D-galactose **#** |
| Allose # | 3-Cyanoalanine # |
| Ascorbate | 4-Hydroxybutyrate # |
| Aspartic acid # | 5-Hydroxyindole-3-acetic acid |
| Carnitine # | Aconitic acid **#** |
| Cholestan-3beta-ol | Allose # |
| Fluorene | alpha-Ketoisocaproic acid |
| Fructose-6-phosphate | Aminomalonic acid |
| Gentiobiose | Androsterone |
| Glucoheptonic acid # | Aspartic acid # |
| Glucose-6-phosphate # | beta-Alanine |
| Glutaraldehyde | beta-Mannosylglycerate |
| Itaconic acid **#** | Carnitine # |
| Lactose # | Cortexolone |
| L-Threose | Cuminic alcohol |
| Maltose | D-Erythronolactone |
| N-Acetyl-D-galactosamine | D-Glyceric acid |
| N-Ethylglycine | Glucoheptonic acid # |
| Serine | Gluconic acid |
|  | Glucose-6-phosphate # |
|  | Itaconic acid **#** |
|  | Lactose # |
|  | L-Malic acid |
|  | Maleimide |
|  | Malonic acid |
|  | N-Acetyl-beta-D-mannosamine |
|  | o-Cresol |
|  | Oxoproline |
|  | Phenyl beta-D-glucopyranoside |
|  | Phenylalanine |
|  | Pyrophosphate |
|  | Sorbitol |
|  | Succinic acid |
|  | Toluenesulfonic acid |
|  | trans-3,5-Dimethoxy-4-hydroxycinnamaldehyde 1 |
|  | Trehalose |
| **CAD vs PMF, Day 4** | **MttA vs PMF, Day 4** |
| 1,5-Anhydroglucitol | 1-Aminocyclopropanecarboxylic acid |
| 2,2-Dimethylsuccinic Acid | 1-Indanone |
| 2,6-Diaminopimelic acid * | 1-Methylhydantoin |
| 2-Amino-1-phenylethanol | 2,6-Diaminopimelic acid * |
| 2-Furoic Acid | 2-Deoxyerythritol |
| 3,6-Anhydro-D-galactose ***** | 2-Hydroxy-3-isopropylbutanedioic acid |
| 3-Hydroxyanthranilic acid * | 2-Mercaptoethanesulfonic acid |
| 4-Aminobutyric acid * | 3,6-Anhydro-D-galactose ***** |
| 5-Aminovaleric acid lactam * | 3-Hydroxyanthranilic acid * |
| Acetol * | 4-Aminobutyric acid * |
| Aconitic acid ***** | 5,6-Dihydrouracil |
| Adenosine * | 5-Aminovaleric acid lactam * |
| Alanine * | 5-Dihydrocortisol |
| alpha-D-Glucosamine 1-phosphate * | 7-alpha-Hydroxycholesterol |
| Azelaic acid | Acetanilide |
| Carnitine * | Acetol * |
| Conduritol b epoxide * | Aconitic acid ***** |
| Cortexolone | Adenosine* |
| D-(Glycerol 1-phosphate) * | Alanine * |
| D-Erythro-sphingosine | Allose |
| d-Glucoheptose * | alpha-D-glucosamine 1-phosphate * |
| D-Glyceric acid * | alpha-Ketoisocaproic acid |
| Digitoxose * | Aminomalonic acid |
| Diglycerol * | Asparagine |
| Elaidic acid * | Aspartic acid |
| Ethanolamine * | beta-Alanine |
| Fumaric acid * | Caprylic acid |
| Gentiobiose * | Carnitine * |
| Glucoheptonic acid | Cellobiose |
| Gluconic acid * | Cellobiotol |
| Glucose-1-phosphate * | Citric acid |
| Glutaraldehyde * | Conduritol b epoxide * |
| Glycine * | Cuminic alcohol |
| Glycolic acid * | D-(Glycerol 1-phosphate) * |
| Inosine | d-Glucoheptose * |
| Isoleucine | D-Glyceric acid * |
| Itaconic acid ***** | Digitoxose * |
| Lactobionic acid | Diglycerol * |
| L-Allothreonine * | Dihydroxyacetone |
| Levoglucosan * | Elaidic acid * |
| L-Malic acid * | Ethanolamine * |
| Lyxonic acid, 1,4-lactone * | Fluorene |
| Maleic acid * | Fumaric acid * |
| Maleimide * | Gentiobiose * |
| Maltose | Gluconic acid * |
| Mannitol * | Glucose-1-phosphate * |
| N-Acetyl-L-leucine * | Glutamic acid |
| N-Cyclohexylformamide * | Glutaraldehyde * |
| Neohesperidin | Glycine * |
| N-Methyl-DL-alanine | Glycolic acid * |
| Oxoproline * | Itaconic acid ***** |
| Palatinose | Lactose |
| Phenylalanine * | L-Allothreonine * |
| Ribitol * | Levoglucosan * |
| Ribose* | Linoleic acid |
| Serine * | L-Malic acid * |
| Sophorose * | L-Threose |
| Sulfuric acid * | Lyxonic acid, 1,4-lactone * |
| Toluenesulfonic acid * | Lyxose |
| Valine * | Maleic acid * |
| Xylitol * | Maleimide * |
| Xylose * | Maltitol |
|  | Mannitol * |
|  | Mannose |
|  | Methyl phosphate |
|  | N-Acetyl-L-leucine * |
|  | N-cyclohexylformamide * |
|  | Norleucine 2 |
|  | O-Acetylserine |
|  | Oleic acid |
|  | O-Phosphorylethanolamine |
|  | Oxoproline * |
|  | Palatinitol |
|  | Palmitic acid |
|  | Palmitoleic acid |
|  | Phenylalanine * |
|  | Progesterone |
|  | Prostaglandin A2 |
|  | Putrescine |
|  | Ribitol * |
|  | Ribonic acid, gamma-lactone |
|  | Ribose * |
|  | Serine * |
|  | Sophorose * |
|  | Sorbose |
|  | Stearic acid |
|  | Succinate semialdehyde |
|  | Sulfuric acid * |
|  | Threitol |
|  | Threonine |
|  | Thymidine |
|  | Toluenesulfonic acid * |
|  | trans-3,5-Dimethoxy-4-hydroxycinnamaldehyde |
|  | Trehalose |
|  | Uracil |
|  | Valine * |
|  | Xylitol * |
|  | Xylose * |

#, common differential metabolites in CAD vs PMF and MttA vs PMF on Day 2, P-value<0.05;

*, common differential metabolites in CAD vs PMF and MttA vs PMF on Day 4, P-value<0.05 and |log_2_FC|>1;

word underline, common differential metabolites under all comparisons.


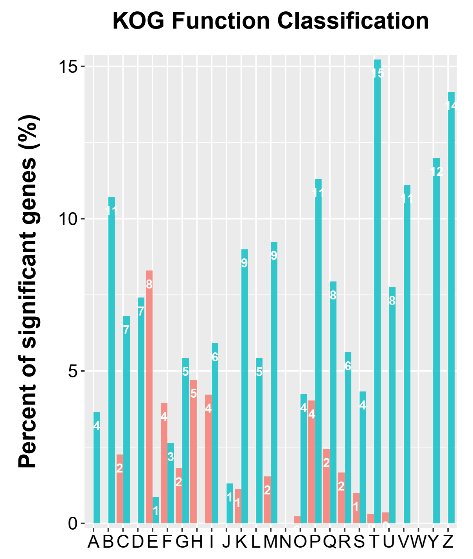

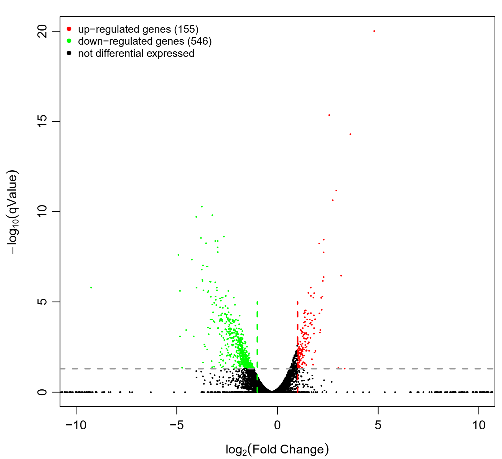
A B


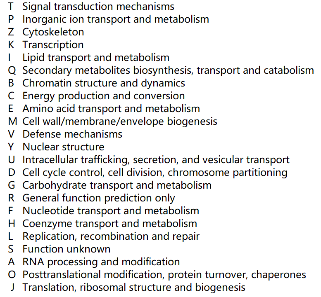

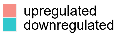


C


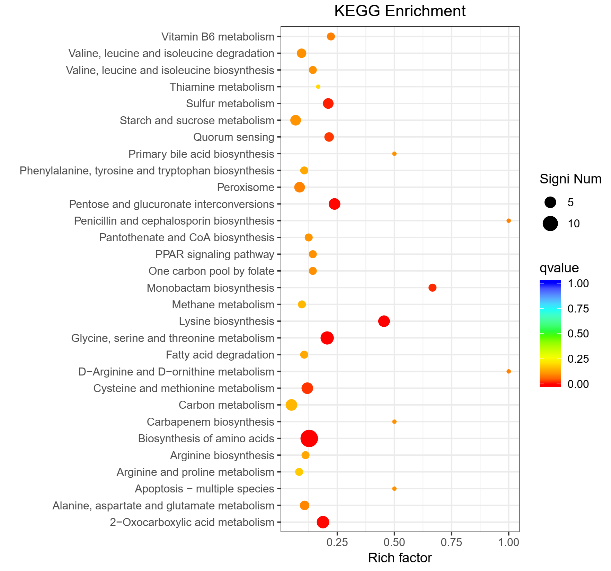

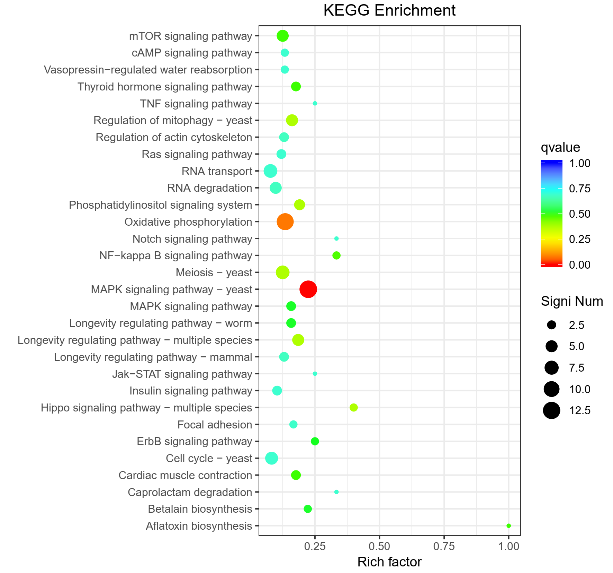


Down-regulated

Up-regulated

**Fig. S4.** (A) Volcano plots of differently expressed genes in MttA vs PMF. Red and green dots respectively denote the upregulated and downregulated differential metabolites (P-value<0.05 and |log_2_FC|>1) in the MttA groups compared with those in the PMF group. KOG (B), and KEGG (C) enrichment analysis of the differently expressed genes in MttA vs PMF.


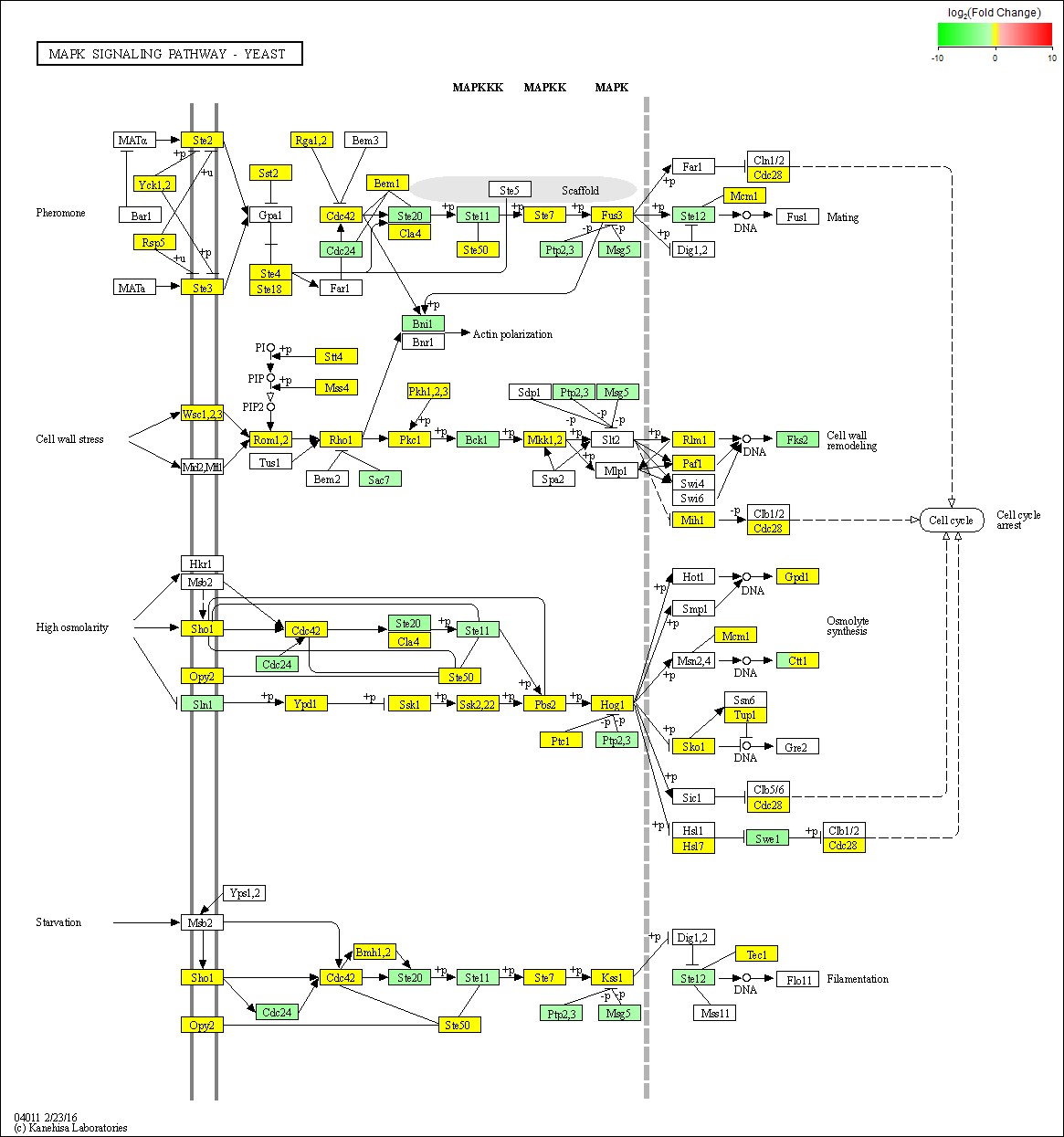


**Fig. S5.** Changes in expression levels of MAPK signaling pathway related genes in MttA compared with PMF.

**Fig. S6.** Comparison of β-glucosidase activities between PMF and MttA strains. *T*-tests were conducted to evaluate statistical significance at p < 0.05(*)

**Table S3. Primers in vector construction.**

| Name | Description |
| --- | --- |
| cad1-F | ctcacatcaaccaaa**tctaga**ATGACCAAGCAGTCCGCCGACT |
| cad1-R | gataagcttgatatc**gaattc**TCAGACGAGGGGGCTCTTG |
| mttA-F | cgcggtggcggccgc**ttaattaa**TCAGTTTGGTTGCGTCAAGAAC |
| mttA-R | ttcacc**accggt**ATGGACTCTAAAATCCAGACAAATGT |
| Pcbh1-F | gagtccat**accggt**GGTGAAGATGAGGCTGAACGG |
| Pcbh1-R | cttctagcggccgc**gcggccgc**CTTGAAGCTGCCAACTCAACC |
| mttAgfp-F | ctcacatcaaccaaa**tctaga**ATGGATAGTAAGATCCAAA |
| mttAgfp-R | ccttgctcaccatg**ttaattaa**GTTAGGCTGGGTGAGGAA |
